# (2*E*)-1-(4,4′′-Difluoro-5′-meth­oxy-1,1′:3′,1′′-terphenyl-4′-yl)-3-[4-(methyl­sulfan­yl)phen­yl]prop-2-en-1-one

**DOI:** 10.1107/S1600536812030139

**Published:** 2012-07-07

**Authors:** Rajni Kant, Vivek K. Gupta, Kamini Kapoor, S. Samshuddin, B. Narayana

**Affiliations:** a X-ray Crystallography Laboratory, Post-Graduate Department of Physics and Electronics, University of Jammu, Jammu Tawi 180 006, India; b Department of Studies in Chemistry, Mangalore University, Mangalagangotri 574 199, India

## Abstract

In the title compound, C_29_H_22_F_2_O_2_S, the central benzene ring makes dihedral angles of 45.83 (7), 38.90 (7) and 55.50 (7)° with the two fluoro-substituted benzene rings and the methyl­sulfanyl-substituted benzene ring, respectively. In the crystal, C—H⋯O contacts connect the mol­ecules into layers lying perpendicular to the *c* axis. In addition, π–π stacking inter­actions between one of the fluoro­phenyl groups [centroid–centroid distances = 3.681 (1) and 3.818 (1) Å] are observed.

## Related literature

For the pharmacological importance of terphenyls, see: Liu (2006 ▶); Gill & Steglich (1987 ▶). For related structures and background to terphenyl chalcones, see: Fun *et al.* (2011 ▶); Fun, Loh *et al.* (2012 ▶); Fun, Hemamalini *et al.* (2012 ▶); Samshuddin *et al.* (2012 ▶).
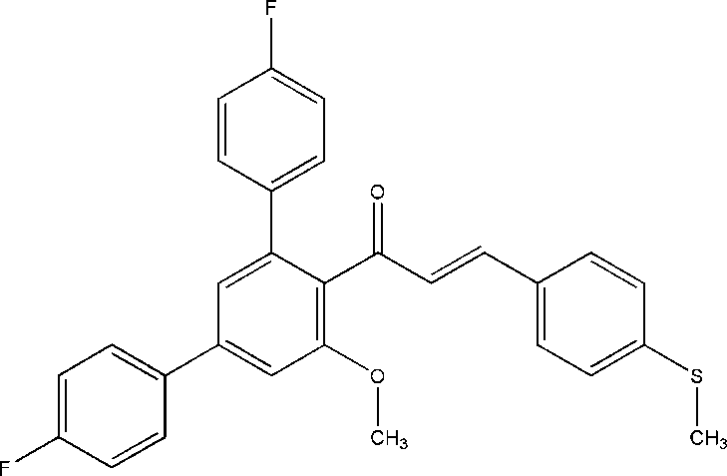


## Experimental

### 

#### Crystal data

C_29_H_22_F_2_O_2_S
*M* *_r_* = 472.53
Triclinic, 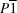

*a* = 6.9341 (3) Å
*b* = 11.4440 (4) Å
*c* = 15.4719 (5) Å
α = 89.611 (3)°
β = 84.738 (3)°
γ = 74.981 (3)°
*V* = 1180.63 (8) Å^3^
*Z* = 2
Mo *K*α radiation
μ = 0.18 mm^−1^
*T* = 293 K
0.3 × 0.2 × 0.1 mm

#### Data collection

Oxford Diffraction Xcalibur Sapphire3 diffractometer
Absorption correction: multi-scan (*CrysAlis PRO*; Oxford Diffraction, 2010 ▶) *T*_min_ = 0.743, *T*_max_ = 1.000
17866 measured reflections
4637 independent reflections
2891 reflections with *I* > 2σ(*I*)
*R*_int_ = 0.044

#### Refinement

*R*[*F* ^2^ > 2σ(*F* ^2^)] = 0.052
*wR*(*F* ^2^) = 0.142
*S* = 1.06
4637 reflections
309 parameters
H-atom parameters constrained
Δρ_max_ = 0.29 e Å^−3^
Δρ_min_ = −0.29 e Å^−3^

### 

Data collection: *CrysAlis PRO* (Oxford Diffraction, 2010 ▶); cell refinement: *CrysAlis PRO*; data reduction: *CrysAlis PRO*; program(s) used to solve structure: *SHELXS97* (Sheldrick, 2008 ▶); program(s) used to refine structure: *SHELXL97* (Sheldrick, 2008 ▶); molecular graphics: *ORTEP-3* (Farrugia, 1997 ▶); software used to prepare material for publication: *PLATON* (Spek, 2009 ▶).

## Supplementary Material

Crystal structure: contains datablock(s) I, global. DOI: 10.1107/S1600536812030139/gk2507sup1.cif

Structure factors: contains datablock(s) I. DOI: 10.1107/S1600536812030139/gk2507Isup2.hkl

Supplementary material file. DOI: 10.1107/S1600536812030139/gk2507Isup3.cml

Additional supplementary materials:  crystallographic information; 3D view; checkCIF report

## Figures and Tables

**Table 1:** Hydrogen-bond geometry (Å, °)

*D*—H⋯*A*	*D*—H	H⋯*A*	*D*⋯*A*	*D*—H⋯*A*
C12—H12⋯O1^i^	0.93	2.47	3.289 (3)	147

Symmetry code: (i) 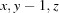.

**Fig. 1.**
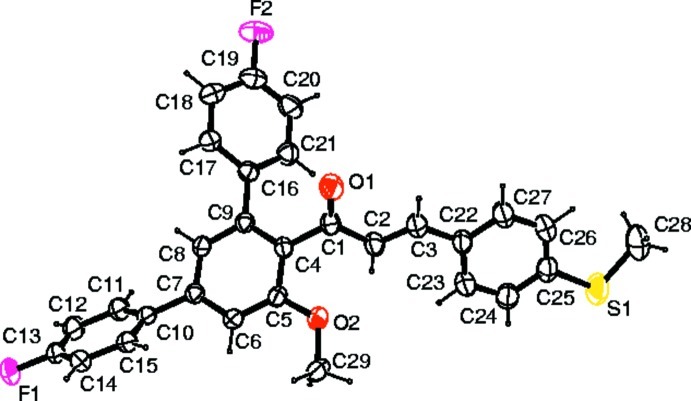
ORTEP view of the molecule with the atom-labeling scheme. The displacement ellipsoids are drawn at the 40% probability level. H atoms are shown as small spheres of arbitrary radii.

**Fig. 2.**
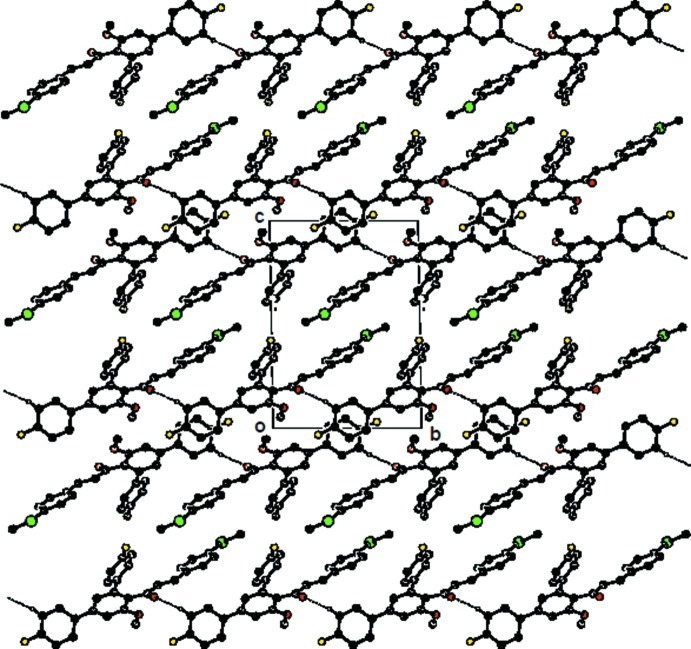
The packing arrangement of molecules viewed down the a axis.

## supplementary crystallographic information

### Comment

The pharmacological importance of terphenyls is well documented (Gill & Steglich, 1987; Liu, 2006). The crystal structure of some terphenyl chalcones, *viz*. (*E*)-1-(4,4''-difluoro-5'-methoxy-1,1':3',1''-terphenyl-4'-yl)-3-(6-methoxy naphthalen-2-yl)prop-2-en-1-one (Fun *et al.*, 2011), (2E)-1- (4,4''-difluoro-5'-methoxy-1,1':3',1''-terphenyl-4'-yl)-3-(2-fluorophenyl) prop-2-en-1-one (Fun, Hemamalini *et al.*, 2012) and (*E*)-3-(2-chlorophenyl) -1-(4,4''-difluoro-5'-methoxy-1,1':3',1''-terphenyl-4'-yl)prop-2-en-1-one (Samshuddin *et al.*, 2012) have been reported. In view of the importance of terphenyls, the title compound (I) was prepared from 4,4'-difluoro chalcone by several steps (Fun, Loh *et al.*, 2012) and its crystal structure is reported here.

All bond lengths and angles are normal and correspond to those observed in related structures. The central benzene ring forms dihedral angles of 45.83 (7), 38.90 (7) and 55.50 (7) ° with the two fluoro- substituted benzene rings and methylsulfanyl substituted benzene ring, respectively. In the crystal, molecules are connected *via* intermolecular C—H···O hydrogen bonds (Fig.2) to form layers. The crystal structure is further stabilized by π-π interactions between the benzene ring (C10—C15) of the molecule at (*x*, *y*, *z*) and the same benzene ring of inversion related molecules at (2 - *x*, -1 - *y*, -*z*) [centroid separation = 3.681 (1) Å, interplanar spacing = 3.512 Å and centroid shift = 1.102 Å] and (1 - *x*, -1 - *y*, -*z*) [centroid separation = 3.818 (1) Å, interplanar spacing = 3.379 Å and centroid shift = 1.777 Å].

### Experimental

To a mixture of 1-(4,4''-difluoro-5'-methoxy-1,1':3',1''-terphenyl-4'-yl)ethanone (0.338 g, 001 mol) and 4-(methylsulfanyl)benzaldehyde (0.152 g, 0.001 mol) in 30 ml ethanol, 0.5 ml of 10% sodium hydroxide solution was added and the reaction mixture was stirred at 5–10°C for 3 hrs. The precipitate formed was collected by filtration and purified by recrystallization from ethanol ( yield 72%; m.p. 407 K). Single-crystal was grown from DMF by slow evaporation method.

### Refinement

All H atoms were positioned geometrically and were treated as riding on their parent C atoms, with C—H distances of 0.93–0.96 Å and with *U*_iso_(H) = 1.2*U*_eq_(C) or 1.5*U*_eq_(methyl C).

### Crystal data

**Table:**

C_29_H_22_F_2_O_2_S	*Z* = 2
*M**_r_* = 472.53	*F*(000) = 492
Triclinic, *P*1	*D*_x_ = 1.329 Mg m^−^^3^
Hall symbol: -P 1	Mo *K*α radiation, λ = 0.71073 Å
*a* = 6.9341 (3) Å	Cell parameters from 6989 reflections
*b* = 11.4440 (4) Å	θ = 3.4–29.0°
*c* = 15.4719 (5) Å	µ = 0.18 mm^−^^1^
α = 89.611 (3)°	*T* = 293 K
β = 84.738 (3)°	Block, colourless
γ = 74.981 (3)°	0.3 × 0.2 × 0.1 mm
*V* = 1180.63 (8) Å^3^	

### Data collection

**Table:**

Oxford Diffraction Xcalibur Sapphire3 diffractometer	4637 independent reflections
Radiation source: fine-focus sealed tube	2891 reflections with *I* > 2σ(*I*)
Graphite monochromator	*R*_int_ = 0.044
Detector resolution: 16.1049 pixels mm^-1^	θ_max_ = 26.0°, θ_min_ = 3.4°
ω scans	*h* = −8→8
Absorption correction: multi-scan (*CrysAlis PRO*; Oxford Diffraction, 2010)	*k* = −14→14
*T*_min_ = 0.743, *T*_max_ = 1.000	*l* = −19→18
17866 measured reflections	

### Refinement

**Table:**

Refinement on *F*^2^	Primary atom site location: structure-invariant direct methods
Least-squares matrix: full	Secondary atom site location: difference Fourier map
*R*[*F*^2^ > 2σ(*F*^2^)] = 0.052	Hydrogen site location: inferred from neighbouring sites
*wR*(*F*^2^) = 0.142	H-atom parameters constrained
*S* = 1.06	*w* = 1/[σ^2^(*F*_o_^2^) + (0.050*P*)^2^ + 0.1774*P*] where *P* = (*F*_o_^2^ + 2*F*_c_^2^)/3
4637 reflections	(Δ/σ)_max_ = 0.001
309 parameters	Δρ_max_ = 0.29 e Å^−^^3^
0 restraints	Δρ_min_ = −0.29 e Å^−^^3^

### Special details

**Table:**

Experimental. *CrysAlis PRO*, Oxford Diffraction Ltd., Version 1.171.34.40 (release 27–08-2010 CrysAlis171. NET) (compiled Aug 27 2010,11:50:40) Empirical absorption correction using spherical harmonics, implemented in SCALE3 ABSPACK scaling algorithm.
Geometry. All e.s.d.'s (except the e.s.d. in the dihedral angle between two l.s. planes) are estimated using the full covariance matrix. The cell e.s.d.'s are taken into account individually in the estimation of e.s.d.'s in distances, angles and torsion angles; correlations between e.s.d.'s in cell parameters are only used when they are defined by crystal symmetry. An approximate (isotropic) treatment of cell e.s.d.'s is used for estimating e.s.d.'s involving l.s. planes.
Refinement. Refinement of *F*^2^ against ALL reflections. The weighted *R*-factor *wR* and goodness of fit *S* are based on *F*^2^, conventional *R*-factors *R* are based on *F*, with *F* set to zero for negative *F*^2^. The threshold expression of *F*^2^ > σ(*F*^2^) is used only for calculating *R*-factors(gt) *etc*. and is not relevant to the choice of reflections for refinement. *R*-factors based on *F*^2^ are statistically about twice as large as those based on *F*, and *R*- factors based on ALL data will be even larger.

### Fractional atomic coordinates and isotropic or equivalent isotropic displacement parameters (Å^2^)

**Table:**

	*x*	*y*	*z*	*U*_iso_*/*U*_eq_	
S1	−0.40890 (12)	0.64035 (8)	0.44889 (6)	0.0889 (3)	
O1	0.7122 (3)	0.17723 (16)	0.18542 (13)	0.0650 (5)	
O2	0.3175 (2)	0.07240 (15)	0.09901 (11)	0.0553 (5)	
F1	0.8319 (2)	−0.69853 (13)	−0.03117 (11)	0.0751 (5)	
F2	1.3029 (2)	−0.00661 (17)	0.42397 (11)	0.0909 (6)	
C1	0.5711 (3)	0.1332 (2)	0.20122 (15)	0.0442 (6)	
C2	0.3756 (3)	0.2042 (2)	0.24325 (15)	0.0480 (6)	
H2	0.2724	0.1664	0.2544	0.058*	
C3	0.3434 (4)	0.3184 (2)	0.26524 (16)	0.0530 (6)	
H3	0.4496	0.3532	0.2521	0.064*	
C4	0.5921 (3)	0.00322 (19)	0.17939 (14)	0.0372 (5)	
C5	0.4622 (3)	−0.0251 (2)	0.12300 (14)	0.0399 (5)	
C6	0.4896 (3)	−0.1416 (2)	0.09174 (14)	0.0395 (5)	
H6	0.4048	−0.1577	0.0528	0.047*	
C7	0.6436 (3)	−0.2346 (2)	0.11840 (14)	0.0380 (5)	
C8	0.7656 (3)	−0.2085 (2)	0.17810 (14)	0.0399 (5)	
H8	0.8633	−0.2714	0.1989	0.048*	
C9	0.7460 (3)	−0.0909 (2)	0.20762 (14)	0.0375 (5)	
C10	0.6860 (3)	−0.3577 (2)	0.08015 (15)	0.0397 (5)	
C11	0.7337 (3)	−0.4601 (2)	0.13080 (17)	0.0497 (6)	
H11	0.7335	−0.4513	0.1905	0.060*	
C12	0.7817 (3)	−0.5751 (2)	0.0935 (2)	0.0566 (7)	
H12	0.8123	−0.6435	0.1276	0.068*	
C13	0.7829 (3)	−0.5854 (2)	0.00604 (19)	0.0508 (7)	
C14	0.7387 (3)	−0.4878 (2)	−0.04658 (17)	0.0508 (6)	
H14	0.7423	−0.4979	−0.1064	0.061*	
C15	0.6882 (3)	−0.3735 (2)	−0.00880 (16)	0.0450 (6)	
H15	0.6552	−0.3060	−0.0436	0.054*	
C16	0.8901 (3)	−0.0692 (2)	0.26791 (14)	0.0403 (5)	
C17	1.0920 (3)	−0.1319 (2)	0.25431 (16)	0.0468 (6)	
H17	1.1337	−0.1881	0.2088	0.056*	
C18	1.2309 (4)	−0.1124 (2)	0.30686 (17)	0.0551 (7)	
H18	1.3654	−0.1544	0.2974	0.066*	
C19	1.1654 (4)	−0.0298 (3)	0.37315 (18)	0.0591 (7)	
C20	0.9699 (4)	0.0323 (3)	0.38994 (17)	0.0588 (7)	
H20	0.9303	0.0883	0.4356	0.071*	
C21	0.8313 (4)	0.0105 (2)	0.33757 (16)	0.0502 (6)	
H21	0.6964	0.0501	0.3495	0.060*	
C22	0.1589 (4)	0.3980 (2)	0.30834 (16)	0.0508 (6)	
C23	−0.0195 (4)	0.3617 (2)	0.32391 (18)	0.0600 (7)	
H23	−0.0242	0.2856	0.3051	0.072*	
C24	−0.1874 (4)	0.4378 (3)	0.36682 (18)	0.0637 (8)	
H24	−0.3044	0.4124	0.3764	0.076*	
C25	−0.1853 (4)	0.5513 (2)	0.39591 (17)	0.0582 (7)	
C26	−0.0107 (4)	0.5880 (3)	0.3797 (2)	0.0715 (8)	
H26	−0.0070	0.6646	0.3979	0.086*	
C27	0.1582 (4)	0.5120 (2)	0.3369 (2)	0.0682 (8)	
H27	0.2743	0.5383	0.3270	0.082*	
C28	−0.3394 (5)	0.7674 (3)	0.4870 (2)	0.0957 (11)	
H28A	−0.2290	0.7409	0.5218	0.144*	
H28B	−0.4512	0.8188	0.5213	0.144*	
H28C	−0.3002	0.8116	0.4384	0.144*	
C29	0.1591 (3)	0.0508 (2)	0.05390 (17)	0.0576 (7)	
H29A	0.2128	0.0144	−0.0018	0.086*	
H29B	0.0621	0.1261	0.0463	0.086*	
H29C	0.0959	−0.0027	0.0870	0.086*	

### Atomic displacement parameters (Å^2^)

**Table:**

	*U*^11^	*U*^22^	*U*^33^	*U*^12^	*U*^13^	*U*^23^
S1	0.0759 (6)	0.0692 (6)	0.1064 (7)	0.0019 (4)	0.0114 (5)	−0.0202 (5)
O1	0.0592 (11)	0.0438 (11)	0.0934 (14)	−0.0207 (9)	0.0062 (10)	0.0036 (10)
O2	0.0536 (10)	0.0405 (10)	0.0678 (12)	0.0007 (8)	−0.0225 (8)	−0.0004 (9)
F1	0.0679 (10)	0.0393 (9)	0.1142 (14)	−0.0066 (7)	−0.0084 (9)	−0.0239 (9)
F2	0.0848 (12)	0.1172 (16)	0.0862 (13)	−0.0433 (11)	−0.0390 (10)	−0.0052 (11)
C1	0.0496 (14)	0.0346 (14)	0.0472 (14)	−0.0087 (11)	−0.0048 (11)	0.0046 (11)
C2	0.0528 (14)	0.0345 (14)	0.0549 (15)	−0.0090 (11)	−0.0030 (11)	0.0009 (12)
C3	0.0559 (15)	0.0407 (15)	0.0605 (17)	−0.0097 (12)	−0.0042 (12)	−0.0028 (13)
C4	0.0384 (12)	0.0306 (12)	0.0412 (13)	−0.0082 (9)	0.0016 (9)	−0.0017 (10)
C5	0.0348 (11)	0.0359 (13)	0.0457 (14)	−0.0037 (10)	−0.0034 (10)	0.0025 (11)
C6	0.0364 (12)	0.0385 (14)	0.0431 (13)	−0.0082 (10)	−0.0059 (10)	0.0010 (11)
C7	0.0358 (12)	0.0339 (13)	0.0438 (13)	−0.0090 (9)	−0.0010 (9)	0.0009 (10)
C8	0.0375 (12)	0.0317 (13)	0.0478 (14)	−0.0035 (9)	−0.0064 (10)	0.0022 (11)
C9	0.0349 (11)	0.0361 (13)	0.0403 (13)	−0.0083 (9)	0.0010 (9)	0.0004 (10)
C10	0.0312 (11)	0.0338 (13)	0.0546 (15)	−0.0082 (9)	−0.0066 (10)	−0.0004 (11)
C11	0.0509 (14)	0.0405 (15)	0.0583 (16)	−0.0124 (11)	−0.0060 (11)	0.0016 (13)
C12	0.0520 (15)	0.0340 (15)	0.084 (2)	−0.0098 (11)	−0.0099 (13)	0.0075 (14)
C13	0.0366 (13)	0.0338 (14)	0.081 (2)	−0.0063 (10)	−0.0062 (12)	−0.0131 (14)
C14	0.0450 (14)	0.0481 (16)	0.0590 (16)	−0.0112 (11)	−0.0055 (11)	−0.0122 (13)
C15	0.0420 (13)	0.0377 (14)	0.0559 (16)	−0.0096 (10)	−0.0097 (11)	0.0001 (12)
C16	0.0423 (12)	0.0379 (13)	0.0424 (13)	−0.0138 (10)	−0.0034 (10)	0.0026 (11)
C17	0.0427 (13)	0.0485 (15)	0.0499 (15)	−0.0131 (11)	−0.0036 (10)	−0.0011 (12)
C18	0.0433 (14)	0.0634 (18)	0.0608 (17)	−0.0160 (12)	−0.0108 (12)	0.0088 (14)
C19	0.0631 (17)	0.068 (2)	0.0560 (17)	−0.0301 (15)	−0.0213 (13)	0.0079 (15)
C20	0.0731 (18)	0.0593 (18)	0.0465 (15)	−0.0198 (14)	−0.0109 (13)	−0.0065 (13)
C21	0.0500 (14)	0.0500 (16)	0.0493 (15)	−0.0111 (11)	−0.0033 (11)	−0.0011 (12)
C22	0.0557 (15)	0.0361 (15)	0.0570 (16)	−0.0041 (11)	−0.0084 (12)	−0.0019 (12)
C23	0.0672 (17)	0.0413 (16)	0.0694 (19)	−0.0096 (13)	−0.0077 (14)	−0.0066 (14)
C24	0.0596 (16)	0.0539 (18)	0.074 (2)	−0.0105 (14)	−0.0006 (14)	−0.0030 (15)
C25	0.0642 (17)	0.0488 (17)	0.0535 (17)	−0.0005 (13)	−0.0042 (12)	−0.0051 (13)
C26	0.078 (2)	0.0461 (18)	0.087 (2)	−0.0097 (15)	−0.0068 (16)	−0.0193 (16)
C27	0.0633 (17)	0.0477 (17)	0.091 (2)	−0.0124 (14)	0.0013 (15)	−0.0157 (16)
C28	0.117 (3)	0.065 (2)	0.090 (3)	−0.0009 (19)	0.004 (2)	−0.020 (2)
C29	0.0407 (13)	0.0608 (18)	0.0663 (18)	−0.0009 (12)	−0.0153 (12)	0.0047 (14)

### Geometric parameters (Å, º)

**Table:**

S1—C25	1.753 (3)	C14—C15	1.382 (3)
S1—C28	1.767 (3)	C14—H14	0.9300
O1—C1	1.217 (3)	C15—H15	0.9300
O2—C5	1.368 (3)	C16—C21	1.378 (3)
O2—C29	1.428 (3)	C16—C17	1.395 (3)
F1—C13	1.367 (3)	C17—C18	1.377 (3)
F2—C19	1.367 (3)	C17—H17	0.9300
C1—C2	1.482 (3)	C18—C19	1.363 (4)
C1—C4	1.494 (3)	C18—H18	0.9300
C2—C3	1.310 (3)	C19—C20	1.360 (4)
C2—H2	0.9300	C20—C21	1.383 (3)
C3—C22	1.470 (3)	C20—H20	0.9300
C3—H3	0.9300	C21—H21	0.9300
C4—C9	1.404 (3)	C22—C27	1.378 (3)
C4—C5	1.406 (3)	C22—C23	1.404 (3)
C5—C6	1.381 (3)	C23—C24	1.377 (3)
C6—C7	1.389 (3)	C23—H23	0.9300
C6—H6	0.9300	C24—C25	1.381 (4)
C7—C8	1.392 (3)	C24—H24	0.9300
C7—C10	1.479 (3)	C25—C26	1.382 (4)
C8—C9	1.393 (3)	C26—C27	1.380 (4)
C8—H8	0.9300	C26—H26	0.9300
C9—C16	1.494 (3)	C27—H27	0.9300
C10—C15	1.387 (3)	C28—H28A	0.9600
C10—C11	1.389 (3)	C28—H28B	0.9600
C11—C12	1.387 (3)	C28—H28C	0.9600
C11—H11	0.9300	C29—H29A	0.9600
C12—C13	1.358 (4)	C29—H29B	0.9600
C12—H12	0.9300	C29—H29C	0.9600
C13—C14	1.364 (3)		
			
C25—S1—C28	103.53 (15)	C21—C16—C9	122.5 (2)
C5—O2—C29	118.40 (19)	C17—C16—C9	119.5 (2)
O1—C1—C2	122.0 (2)	C18—C17—C16	121.4 (2)
O1—C1—C4	120.2 (2)	C18—C17—H17	119.3
C2—C1—C4	117.7 (2)	C16—C17—H17	119.3
C3—C2—C1	122.1 (2)	C19—C18—C17	118.0 (2)
C3—C2—H2	118.9	C19—C18—H18	121.0
C1—C2—H2	118.9	C17—C18—H18	121.0
C2—C3—C22	127.5 (2)	C20—C19—C18	122.9 (2)
C2—C3—H3	116.2	C20—C19—F2	118.5 (3)
C22—C3—H3	116.2	C18—C19—F2	118.5 (2)
C9—C4—C5	118.7 (2)	C19—C20—C21	118.5 (2)
C9—C4—C1	122.53 (19)	C19—C20—H20	120.7
C5—C4—C1	118.6 (2)	C21—C20—H20	120.7
O2—C5—C6	124.1 (2)	C16—C21—C20	121.0 (2)
O2—C5—C4	114.4 (2)	C16—C21—H21	119.5
C6—C5—C4	121.4 (2)	C20—C21—H21	119.5
C5—C6—C7	120.0 (2)	C27—C22—C23	117.6 (2)
C5—C6—H6	120.0	C27—C22—C3	120.0 (2)
C7—C6—H6	120.0	C23—C22—C3	122.4 (2)
C6—C7—C8	118.8 (2)	C24—C23—C22	120.6 (2)
C6—C7—C10	120.8 (2)	C24—C23—H23	119.7
C8—C7—C10	120.2 (2)	C22—C23—H23	119.7
C7—C8—C9	122.0 (2)	C23—C24—C25	121.2 (3)
C7—C8—H8	119.0	C23—C24—H24	119.4
C9—C8—H8	119.0	C25—C24—H24	119.4
C8—C9—C4	118.9 (2)	C24—C25—C26	118.4 (2)
C8—C9—C16	118.8 (2)	C24—C25—S1	117.0 (2)
C4—C9—C16	122.3 (2)	C26—C25—S1	124.6 (2)
C15—C10—C11	118.3 (2)	C27—C26—C25	120.7 (3)
C15—C10—C7	120.1 (2)	C27—C26—H26	119.7
C11—C10—C7	121.5 (2)	C25—C26—H26	119.7
C12—C11—C10	120.9 (2)	C22—C27—C26	121.6 (3)
C12—C11—H11	119.5	C22—C27—H27	119.2
C10—C11—H11	119.5	C26—C27—H27	119.2
C13—C12—C11	118.4 (3)	S1—C28—H28A	109.5
C13—C12—H12	120.8	S1—C28—H28B	109.5
C11—C12—H12	120.8	H28A—C28—H28B	109.5
C12—C13—C14	122.9 (2)	S1—C28—H28C	109.5
C12—C13—F1	118.7 (2)	H28A—C28—H28C	109.5
C14—C13—F1	118.4 (2)	H28B—C28—H28C	109.5
C13—C14—C15	118.3 (2)	O2—C29—H29A	109.5
C13—C14—H14	120.9	O2—C29—H29B	109.5
C15—C14—H14	120.9	H29A—C29—H29B	109.5
C14—C15—C10	121.2 (2)	O2—C29—H29C	109.5
C14—C15—H15	119.4	H29A—C29—H29C	109.5
C10—C15—H15	119.4	H29B—C29—H29C	109.5
C21—C16—C17	118.1 (2)		
			
O1—C1—C2—C3	0.4 (4)	C12—C13—C14—C15	−0.7 (3)
C4—C1—C2—C3	179.6 (2)	F1—C13—C14—C15	179.68 (18)
C1—C2—C3—C22	−178.9 (2)	C13—C14—C15—C10	1.2 (3)
O1—C1—C4—C9	53.5 (3)	C11—C10—C15—C14	−0.7 (3)
C2—C1—C4—C9	−125.7 (2)	C7—C10—C15—C14	176.28 (19)
O1—C1—C4—C5	−121.5 (2)	C8—C9—C16—C21	−140.0 (2)
C2—C1—C4—C5	59.3 (3)	C4—C9—C16—C21	40.3 (3)
C29—O2—C5—C6	13.8 (3)	C8—C9—C16—C17	39.7 (3)
C29—O2—C5—C4	−169.76 (19)	C4—C9—C16—C17	−140.0 (2)
C9—C4—C5—O2	−179.93 (18)	C21—C16—C17—C18	−2.1 (4)
C1—C4—C5—O2	−4.7 (3)	C9—C16—C17—C18	178.2 (2)
C9—C4—C5—C6	−3.4 (3)	C16—C17—C18—C19	0.1 (4)
C1—C4—C5—C6	171.84 (19)	C17—C18—C19—C20	0.9 (4)
O2—C5—C6—C7	178.6 (2)	C17—C18—C19—F2	−177.8 (2)
C4—C5—C6—C7	2.4 (3)	C18—C19—C20—C21	0.2 (4)
C5—C6—C7—C8	1.3 (3)	F2—C19—C20—C21	178.9 (2)
C5—C6—C7—C10	−174.82 (19)	C17—C16—C21—C20	3.2 (4)
C6—C7—C8—C9	−4.1 (3)	C9—C16—C21—C20	−177.2 (2)
C10—C7—C8—C9	172.08 (19)	C19—C20—C21—C16	−2.3 (4)
C7—C8—C9—C4	3.1 (3)	C2—C3—C22—C27	171.7 (3)
C7—C8—C9—C16	−176.63 (19)	C2—C3—C22—C23	−6.8 (4)
C5—C4—C9—C8	0.7 (3)	C27—C22—C23—C24	−0.5 (4)
C1—C4—C9—C8	−174.35 (19)	C3—C22—C23—C24	178.1 (2)
C5—C4—C9—C16	−179.65 (19)	C22—C23—C24—C25	−0.2 (4)
C1—C4—C9—C16	5.3 (3)	C23—C24—C25—C26	1.0 (4)
C6—C7—C10—C15	43.8 (3)	C23—C24—C25—S1	179.5 (2)
C8—C7—C10—C15	−132.3 (2)	C28—S1—C25—C24	173.2 (2)
C6—C7—C10—C11	−139.4 (2)	C28—S1—C25—C26	−8.4 (3)
C8—C7—C10—C11	44.6 (3)	C24—C25—C26—C27	−1.1 (4)
C15—C10—C11—C12	−0.2 (3)	S1—C25—C26—C27	−179.5 (2)
C7—C10—C11—C12	−177.2 (2)	C23—C22—C27—C26	0.4 (4)
C10—C11—C12—C13	0.7 (3)	C3—C22—C27—C26	−178.2 (3)
C11—C12—C13—C14	−0.2 (4)	C25—C26—C27—C22	0.4 (5)
C11—C12—C13—F1	179.42 (19)		

### Hydrogen-bond geometry (Å, º)

**Table:**

*D*—H···*A*	*D*—H	H···*A*	*D*···*A*	*D*—H···*A*
C12—H12···O1^i^	0.93	2.47	3.289 (3)	147

Symmetry code: (i) *x*, *y*−1, *z*.
